# Description of two new species of *Diplectanum* Diesing, 1858 (Monogenea: Diplectanidae) collected from *Protonibea diacanthus* (Lacepède, 1802) (Teleostei: Sciaenidae) from waters off northern Australia

**DOI:** 10.1016/j.ijppaw.2023.04.004

**Published:** 2023-04-17

**Authors:** Megan Porter, Diane P. Barton, Nidhish Francis, Shokoofeh Shamsi

**Affiliations:** a School of Agricultural, Environmental and Veterinary Sciences, Charles Sturt University, Wagga Wagga, NSW, 2678, Australia; b Gulbali Institute, Charles Sturt University, Wagga Wagga, NSW, 2678, Australia

**Keywords:** Diplectanidae, Sciaenidae, Monogenean, Phylogenetic analysis, Taxonomy

## Abstract

Two new species of the family Diplectanidae Monticelli, 1903 from the gills of *Protonibea diacanthus* (Lacepède, 1802) (Teleostei: Sciaenidae) off the northern Australian coast are described. Previous studies have either morphological or genetic results, whereas this study combines morphological and advanced molecular methods to provide the first detailed descriptions for species of *Diplectanum* Diesing, 1858 from Australia utilising both methodologies. Two new species, *Diplectanum timorcanthus* n. sp. and *Diplectanum diacanthi* n. sp., are morphologically described and genetically characterised using the partial nuclear 28S ribosomal RNA gene (28S rRNA) and the internal transcribed spacer 1 (ITS1) partial sequence.

## Highlights

• Species of *Diplectanum* Diesing, 1858 (Monogenea: Diplectanidae) infect sciaenid hosts in Australia.
• Phylogeny helps to understand evolutionary relationships of monogenean taxonomy.
• Combined morphological and molecular descriptions of two new species of gill parasite in Australia are provided.

## Graphical abstract

**Figure:**
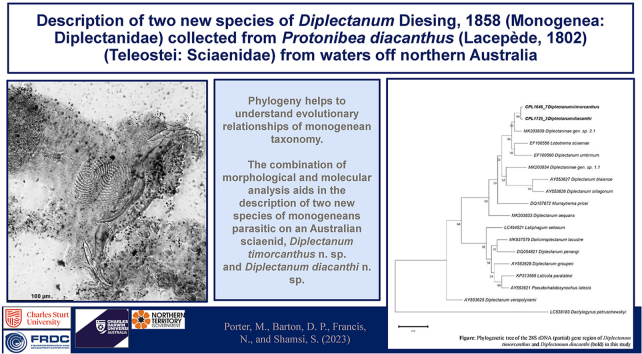

## 1 Introduction

Belonging to the order Eupercaria *incertae sedis*, fishes of the family Sciaenidae Cuvier, 1829 are widely distributed in the Atlantic, Indian, and Pacific oceans. *Protonibea diacanthus* (Lacepède, 1802)*,* the black-spotted croaker, is the largest genus of the Sciaenidae in Australian waters and is found both in inshore and nearshore estuarine and coastal waters of Australia (Froese and Pauly 2023). This species is distributed from Hervey Bay in Queensland across the northern coastline to Shark Bay in Western Australia and is considered a significant contributor to the recreational, traditional, and commercial fishing sectors of Australia (Taillebois et al., 2017). *Protonibea diacanthus’* sexual maturity is reached at age 2+ with an expected lifespan of 13 years (Phelan et al., 2008). The species grows to a significant size, with reports exceeding 150 cm in length and 45 kg in weight (Phelan et al., 2008). Being a sedentary predatory fish, the species is easily targeted by consumers, and for this reason the *P. diacanthus* fishery is one of the biggest off northern Australia. *Protonibea diacanthus*, like all other sciaenid species, is challenged by overfishing and evolving environmental changes, but despite growing pressures on the commercially important sciaenid, there remains to be limited knowledge on the key parasites hosted by *P. diacanthus,* and the potential effects that these may have on the fish host.

Monogenean ectoparasites belonging to the family Diplectanidae Monticelli, 1903 are represented by over 250 species primarily infecting the gills of marine perciform fishes (Domingues and Boeger 2008). Within the Diplectanidae, *Diplectanum* Diesing, 1858 is a diverse genus of monopisthocotylean monogeneans which currently has 28 nominal species recognised as parasites of perciform fishes (Villar-Torres et al., 2019; WoRMS 2023). Species of *Diplectanum* show variable levels of host specificity with a small number showing high specificity but the majority being generalist. There has been a number of reports coming from both freshwater and marine sciaenids in Brazil (Boeger et al., 2006; Domingues and Boeger 2003), however the most common reports of species of *Diplectanum* have been amongst Mediterranean sciaenids. Despite the numerous worldwide reports of *Diplectanum* spp., reports in Australian Sciaenidae are scarce. *Diplectanum oliveri* Williams (1989) and *Diplectanum glandulosum* Williams (1989) were described from the marine host *Argyrosomus hololepidotus* (Lacepède, 1801) (Sciaenidae) in Western Australia (Williams 1989). The only other reports of species of *Diplectanum* in Australia came from Young (1969) who described four new species from serranid fishes in Queensland, of which all have since been transferred to different genera (Justine and Euzet 2006; Kritsky and Beverley-Burton 1986).

As with many species of monogeneans, differentiation is most often solely by morphological characteristics, in particular the morphology of the copulatory and haptoral structures. Molecular study of species within the genus *Diplectanum* is scarce (Villar-Torres et al., 2019), with many specimens not morphologically characterised or identified to species (Domingues and Boeger 2008). Improved descriptions with a combination of morphological and genetic analysis, are required so that the validity of the many species of *Diplectanum* will be confirmed (Villar-Torres et al., 2019). As such, the present study contributes the first detailed report of monogenean parasites from *P. diacanthus* in Australian waters and describes two new species of *Diplectanum* using both molecular and morphological identification techniques.

## 2 Materials and methods

### 2.1 Fish collection

A total of 228 *Protonibea diacanthus* were collected off the coast of the Northern Territory of Australia, during 2020 and 2021. Fish were collected using hook and line capture and euthanised via percussive stunning. Fish dissection was performed on fresh samples, with the gills removed, bagged, and stored frozen for later processing.

### 2.2 Parasite collection

Gills from each fish were examined for the presence of monogeneans. Once thawed, bone-cutters were used to separate gill arches prior to washing. Separated gill arches were placed in a large jar of water and shaken vigorously to remove any parasitic organisms. Gill arches were removed from the jar and gill lamellae thoroughly inspected for parasites under a Leica EZ4 dissecting microscope. The remaining gill wash was allowed to settle, and the supernatant poured off, leaving the sediment behind. The sediment was examined under a Leica EZ4 dissecting microscope for the identification and collection of parasites. Monogeneans were removed from the gill wash using a small probe and preserved in 70% Ethanol until later processing.

### 2.3 Morphological examination

Monogeneans were mounted on a microscope slide with 70% ethanol and 1 drop of acetostain, before a coverslip was placed. The coverslip edges were then secured with glycerine jelly. Measurements, all in micrometres (μm), of characters of systemic importance were obtained with the use of a Nikon DS-Ri2 motorised microscope, and are given as the range, followed by the mean in parentheses (Table 2). A dash (−) indicates that measurements could not be made or were not available. Electronic images of specimens were captured using the motorised microscope and appropriate scales are provided. Drawings of important morphological characteristics were completed with the use of a Nikon Y-IDT drawing tube which was mounted on a Nikon Eclipse E200 microscope.

**Table 1:** Details of the sequences used in the present study to construct the phylogenetic trees based on 28S and ITS1 data.

Monogenean species	Host species	Host family	Geographical origin	GenBank ID		Reference
				28S	ITS1	
*Diplectanum timorcanthus* n. sp.	*Protonibea diacanthus*	Sciaenidae	Australia: Northern Territory	XXXX	XXXX	Present study
*Diplectanum diacanthi* n. sp.	*Protonibea diacanthus*	Sciaenidae	Australia: Northern Territory	XXXX	XXXX	Present study
Diplectanidae sp. 2.1	*Umbrina canariensis*	Sciaenidae	La Ràpita and Santa Pola: Spain	MK203838	MK208310	Villar-Torres et al., (2019)
*Lobotrema sciaenae*		Sciaenidae	Indo-Pacific	EF100556		Wu et al. unpublished
*Diplectanum umbrinum inc. sed.*		Sciaenidae	Indo-Pacific	EF100560		Wu et al. unpublished
*Diplectanum aequans*	*Dicentrarchus labrax*	Moronidae	La Ràpita: Spain	MK203833		Villar-Torres et al., (2019)
Diplectanidae sp. 1.1	*Argyrosomus regius*	Sciaenidae	Burriana: Spain	MK203834		Villar-Torres et al., (2019)
*Diplectanum blaiense*^a^	*Sillago sihama*	Sillaginidae	China: Hainan	AY553627		Wu et al., (2005)
*Diplectanum sillagonum*^b^	*Sillago sihama*	Sillaginidae	China: Hainan	AY553626		Wu et al., (2005)
*Diplectanum veropolynemi*	*Polynemus sextarius*	Polynemidae	China: Huiyang	AY553625		Wu et al., (2005)
*Latiphagum setosum*	*Psammoperca waigiensis*	Latidae	Okinawa-jima Island: Japan	LC494521		Nitta (2019)
*Laticola paralatesi*	*Lates calcarifer*	Latidae	Townsville QLD: Australia	KP313568		Chotnipat et al., (2015)
*Pseudorhabdosynochus latesis*^c^	*Lates calcarifer*	Latidae	China: Yangjiang	AY553621		Wu et al., (2005)
*Murraytrema pricei*	*Nibea albiflora*	Sciaenidae	Guangdong Province: Panyu	DQ157672		Wu et al., (2006)
*Dolicirroplectanum lacustre*	*Lates niloticus*	Latidae	Lake Volta: Ghana, and Lake Albert: Uganda	MK937579		Kmentová et al., 2020
*Diplectanum grouperi*^d^	*Epinephelus coioides*	Serranidae	China: Huidong	AY553628		Wu et al., (2005)
*Diplectanum penangi*^e^	*Lates calcarifer*	Latidae	South China Sea	DQ054821		Yang et al. (2006)
*Dactylogyrus petruschewskyi*	*Megalobrama amblycephala*	Cyprinidae	Ono River, Inashiki City, Ibaraki Prefecture.	LC538183		Nitta et al., (2020)
*Diplectanum aequans*	*Dicentrarchus labrax*	Moronidae	Black Sea: Turkey		MH400167	Aydin and Pekmezci (2022)
*Dolicirroplectanum lacustre*	*Lates perches*	Latidae	African Great Lakes		MK937576	Kmentová et al., 2020
*Dolicirroplectanum lacustre*	*Lates niloticus*	Latidae	Lake Albert and Lake Victoria: Uganda		OP422433	Thys et al., (2022)
*Sciadicleithrum* sp. 3	*Crenicichla lenticulata*	Cichlidae	Nergo River: Brazil		ON368058	Seidlová et al., (2022)
*Gussevia astronoti*	*Crenicichla lenticulata*	Cichlidae	Nergo River: Brazil		ON368042	Seidlová et al., (2022)
*Gussevia asota*	*Astronotus ocellatus*	Cichlidae	Amazon River: Peru		ON368041	Seidlová et al., (2022)
*Sciadicleithrum umbilicum*	*Cichla monoculus*	Cichlidae	Amazon River: Peru		ON368065	Seidlová et al., (2022)
*Dactylogyrus formosus*	*Carassius gibelio*	Cyprinidae	Baštica reservoir: Baštica		MG792869	Benovics et al., (2018)
*Dactylogyrus anchoratus*	*Carassius gibelio*	Cyprinidae	Baštica reservoir: Baštica		KY859795	Benovics et al., (2018)
*Dactylogyrus formosus*	*Carassius auratus*	Cyprinidae	Henan Province: China		KM525669	Tu et al., (2015)
*Thaparocleidus siluri*	*Silurus glanis*	Siluridae	Morava basin: Czech Republic		AJ490164	Simková et al. (2003)
*Sciadicleithrum ergensi*	*Cichla monoculus*	Cichlidae	Amazon River: Peru		ON368052	Seidlová et al., (2022)
*Dactylogyrus vastator*	*Carassius gibelio*	Cyprinidae	River Dyje: Czech Republic		KY201103	Benovics et al., (2017)
*Benedenia seriolae*	*Seriola quinqueradiata*	Carangidae	Hyogo: Japan		LC718918	Nitta (2023)

a Redescribed as *Paradiplectanum blairense* (Gupta and Khanna, 1974) Domingues and Boeger (2008).

b Redescribed as *Paradiplectanum sillagonum* (Tripathi, 1959) Domingues and Boeger (2008).

c Redescribed as *Laticola latesi* (Tripathi, 1959) Yang et al. (2006).

d Redescribed as *Pseudorhabdosynochus grouperi* (Bu, Leong, Wong, Woo & Foo, 1999) Wu et al., 2005.

e Redescribed as *Dolicirroplectanum penangi* (Liang and Leong, 1991) Kmentová et al., 2020

**Table 2:** Comparative measurements of *Diplectanum* spp. (measurements in micrometres; measurements are range followed by mean in parentheses; - indicates no measurements available.).

**Species**	***Diplectanum timorcanthus* n. sp**.	***Diplectanum diacanthi* n. sp**.	***Diplectanum oliveri***	***Diplectanum glandulosum***
**Reference**	Present study	Present study	Williams (1989)	Williams (1989)
**Host**	*Protonibea diacanthus*	*Protonibea diacanthus*	*Argyrosomus hololepidotus*	*Argyrosomus hololepidotus*
**Location**	Australia: Northern Territory	Australia: Northern Territory	Australia: Western Australia	Australia: Western Australia
Total length	546-1830 (923)	645-1870 (1087)	909-1139 (1010)	499-640 (594)
Greatest width	75-214 (145)	127-460 (253)	166-224 (195)^a^	128-154 (139)^a^
Eye spots	2 anterior to pharynx	2 anterior to pharynx	2 anterior to pharynx	large, intact
Tegumental scales	absent	absent	absent	absent
Pharynx Length	38-79 (56)	38-101 (64)	–	–
Pharynx Width	34-60 (46)	27-95 (62)	41-71 (56)	35-51 (39)
Peduncle Length	57-212 (133)	89-366 (168)	–	–
Peduncle Width	35-147 (75)	54-163 (96)	–	–
Haptor Length	37-94 (64)	51-101 (67)	–	–
Haptor Width	191-291 (226)	151-301 (235)	224-275 (258)	243-269 (254)
Oral Sucker Length	8-18 (13)	10-24 (15)	–	–
Oral Sucker Width	8-21 (12)	9-21 (13)	–	–
Squamodisc Length	64-164 (118)	23-74 (48)	–	–
Squamodisc Width	34-156 (105)	35-101 (65)	166-189 (175)	99-106 (101)
Ventral Anchor Length	56-67 (61)	46-75 (60)	67-77 (72)	72-77 (74)
Ventral Anchor Base Width	2-15 (8)	4-20 (10)	–	–
Dorsal Anchor Length	52-69 (62)	46-78 (66)	66-70 (68)	64-67 (65)
Dorsal Anchor Base Width	6-12 (9)	4-14 (10)	–	–
Ventral Bar Length	98-133 (118)	93-124 (109)	128-158 (148)	117-153 (134)
Ventral Bar Width	9-26 (21)	8-19 (16)	–	–
Dorsal Bar Length	64-108 (97)	48-66 (61)	96-110 (102)	90-117 (105)
Dorsal Bar Width	14-28 (23)	12-40 (20)	–	–
MCO Length	42-96 (74)	81-110 (96)	166-179 (171)	51-66 (59)
MCO Width	5-15 (10)	9-12 (10)	–	–

a At level of ovary.

### 2.4 Molecular sequencing

The monogenean parasites were separated into morphotypes based on overall morphological appearance, and those chosen for molecular study were collected and transferred into 1.5 ml autoclaved Eppendorf tubes. Due to the microscopic size of the organisms, entire individual specimens were used for molecular processing. Genomic DNA was extracted using DNeasy Blood & Tissue Kits (Qiagen), according to the modified protocol of the manufacturer's guidelines (Shamsi et al., 2018) and eluted in 40 μl of elution buffer. Polymerase Chain Reaction (PCR) amplification of the nuclear 28S rDNA (partial) gene was carried out using the forward primer C1 (5ʹ-ACCCGCTGAATTTAAGCAT-3ʹ) and reverse primer D2 (5ʹ-TCCGTGTTTCAAGACGG-3ʹ) (Villar-Torres et al., 2019). Polymerase Chain Reaction (PCR) amplification was also carried out for the nuclear ITS1 region using the forward primer S1 (5ʹ- ATTCCGATAACGAACGAGACT -3ʹ) and reverse primer IR8 (5ʹ-GCTAGCTGCGTTCTTCATCGA -3ʹ) (Wu et al., 2005). Each amplification reaction contained 4 μl template DNA, 5 μl 5X GoTaq® Flexi Buffer, 2.5 μl MgCl_2_, 1 μl dNTP at 10 mM, 0.5 μl of each primer at 10 μM and 0.25 μl GoTaq® Flexi DNA Polymerase in a total volume of 25 μl. The PCR cycling conditions to amplify the nuclear 28S rDNA (partial) gene was carried out with the following steps: initial denaturation at 94 °C for 5 min, followed by 30 cycles of amplification: denaturation at 94 °C for 1 min, annealing at 56 °C for 1 min, primer extension at 72 °C for 1 min; and a final extension at 72 °C for 10 min. The PCR cycling conditions to amplify the nuclear ITS1 gene region was carried out with the following steps: initial denaturation at 95 °C for 4 min, followed by 40 cycles of amplification: denaturation at 92 °C for 1 min, annealing at 55 °C for 1 min, primer extension at 72 °C for 1 min 30 s; and a final extension at 72 °C for 10 min. An aliquot (2.5 μl) of each amplicon was examined on a 1.5% w/v agarose gel, stained with GelRed™ and photographed upon transillumination.

Representative samples were sent to the Australian Genome Research Facility (AGRF) and were subjected to Sanger sequencing using the same primer sets used for PCR. Forward and reverse AB1 trace files (chromatograms) were quality-checked using Sequence Scanner Software 2 (Applied Biosystems/Thermo Fisher). Subsequently, sequences were aligned by MUSCLE using MEGA version 11 (Tamura et al., 2021), followed by manual adjustment.

Slide-mounted and unmounted specimens were deposited in the collections of the Museum and Art Gallery of the Northern Territory (MAGNT), Queensland Museum (QM), and the Western Australian Museum (WAM). The sequencing data resulting from this study were deposited for 28S, under GenBank accession numbers OQ846930-OQ846931, and for ITS1, under GenBank accession numbers OQ846935-OQ846937.

### 2.5 Construction of phylogenetic tree

Phylogenetic trees for both gene regions (28S rDNA (partial) and ITS1) were constructed from the sequences generated in this study along with representative (similar and closely related species) sequences from GenBank (Table 1), and two outgroups for both the 28S rDNA (partial) region and the ITS1 region, respectively. After alignment and manual adjustment, phylogenetic trees were constructed using the Maximum Likelihood analysis, Tamura-Nei model in MEGA version 11 (Tamura et al., 2021). The reliability of the Maximum Likelihood Tree was assessed by the Bootstrap method, with 1000 replications.

## 3 Results

### 3.1 Taxonomy

**Class Monogenea** Bychowsky, 1937

**Subclass Monopisthocotylea** Bychowsky, 1937

**Order Dactylogyride**a Bychowsky, 1937

**Family Diplectanidae** Monticelli, 1903

**Genus *Diplectanum*** Diesing, 1858

**Fig. 1:**
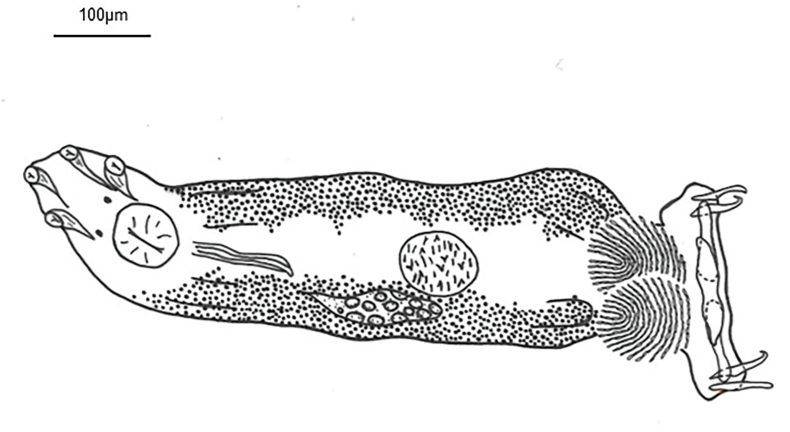
Composite drawing of *Diplectanum timorcanthus* n. sp.

**Fig. 2:**
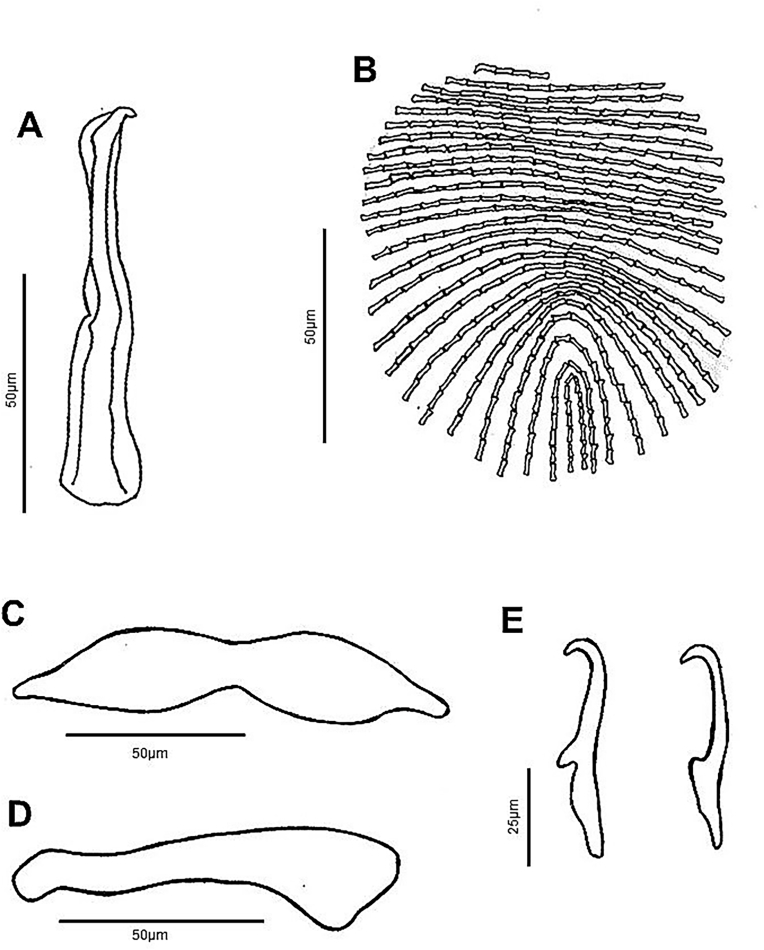
*Diplectanum timorcanthus* n. sp. characters of morphological significance. A, MCO; B, Squamodisc; C, Ventral Bar; D, Dorsal Bar; E, Ventral and Dorsal Anchors.

**Fig. 3:**
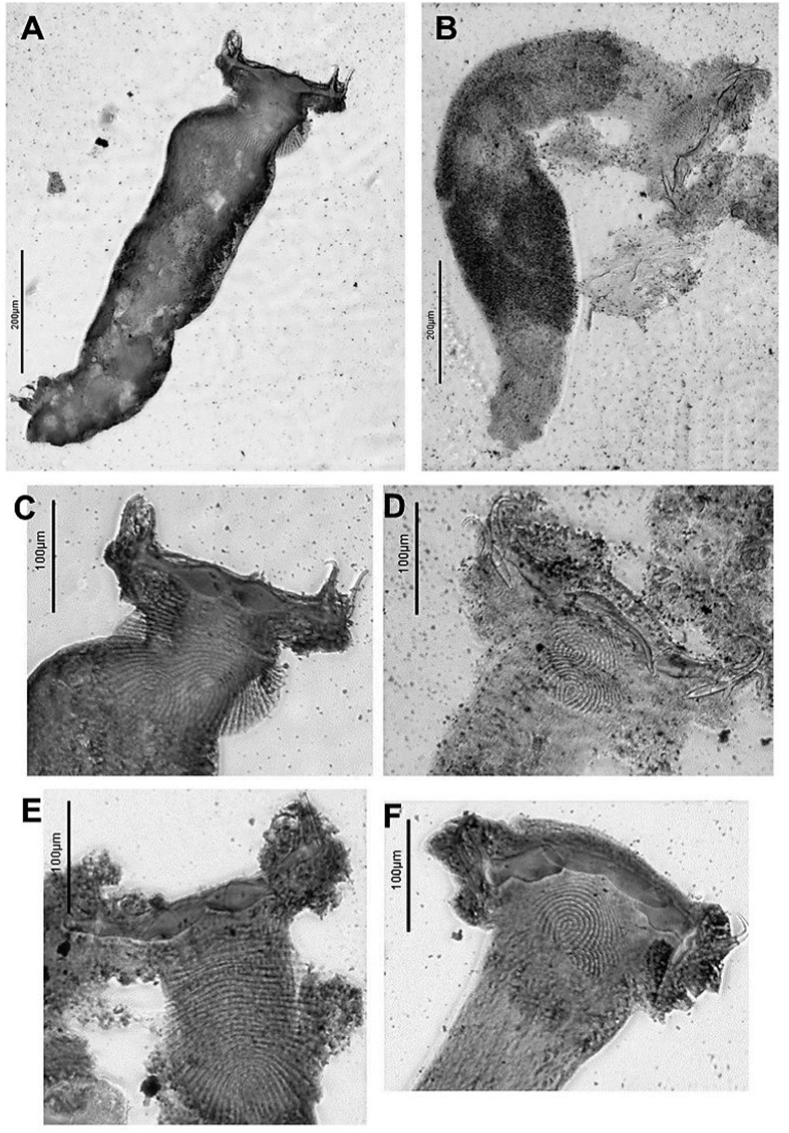
*Diplectanum timorcanthus* n. sp. A, Full body; C and E, Haptoral structures. *Diplectanum diacanthi* n. sp. B, Full body; D and F, Haptoral structures.

***Diplectanum timorcanthus* n. sp.** (Fig. 1; Fig. 2; Fig. 3A, C, E)

*Type host: Protonibea diacanthus* (Lacepède, 1802) (Sciaenidae) ‘Black Jewfish’ or ‘Black-spotted croaker’.

*Type locality:* Caution Point, Beagle Gulf, Northern Territory, Australia. Other localities include Van Diemen Gulf, Timor Sea, Northern Territory, Australia.

*Site in host:* Gill filaments.

*Type-material:* Holotype: MAGNT D001920; Paratypes: MAGNT D001921, QM G240632, WAM V11009.

*GenBank accession:* 28S (OQ846930-OQ846931), ITS1 (OQ846935-OQ846937).

*Etymology:* The specific name resembles a combination of the geographical location, and the host species, from which the type specimen of *Diplectanum timorcanthus* n. sp. was collected.

#### 3.1.1 Description

Based on 39 specimens. Body elongate, fusiform, with smooth tegument; total length 923 (546–1,830); greatest width 145 (75-214), near level of testis. Four cephalic lobes moderately defined. Two pairs of head organs, 13 (8-18) long, 12 (8-21) wide, moderately developed as cephalic lobes, adjacent cephalic areas. Cephalic glands poorly defined, extending to pharyngeal region. Eye spots 2 when visible, poorly developed, or represented by scattered granules, dorsal, anterior to pharynx. Mouth subterminal; pharynx bulbous, length 56 (38-79), width 46 (34-60). Oesophagus short to absent. Intestinal caeca bifurcates short distance posterior to pharynx, crura blind posteriorly. Peduncle slightly tapered posteriorly, 133 (57-212) long, 75 (35-147) wide, peduncular spines absent. Haptor laterally expanded (Figs. 3C), 64 (37-94) long, 226 (191-291) wide. Squamodiscs ovate, ventral and dorsal (Fig. 3C and E); each formed with approximately 25 rows of sclerotized dumbbell-shaped rods (Fig. 2B); squamodisc 118 (64-164) long, 105 (34-156) wide. Anchors 2 pairs, ventral and dorsal (Fig. 2E). Ventral anchor length 61 (56-67), anchor base width 8 (2-15); anchor with elongate deep root longer than superficial root, knob-like superficial root, curved shaft, short point (Fig. 2E). Dorsal anchor length 62 (52-69), anchor base width 9 (6-12); anchor with wide tapered deep root, incipient superficial root, straight shaft, short point (Fig. 2E). Hooks not observed. Ventral bar with tapering ends, constricted midregion, ventral longitudinal groove (Figs. 2C), 118 (98-133) long, greatest width 21 (9-26). Paired dorsal bar, 97 (64-108) long, greatest width 23 (14-28); not overlapping each other; medial end expanding wider than lateral end, medial end overlapping ventral bar extremities (Figs. 1 and 2D). Male copulatory organ (MCO) sclerotized, elongate (Figs. 2A), 74 (42-96) long, greatest width 10 (5-15); composed of two nested tubes which appear to be joined at the proximal end. Slightly curved at distal end, inner tube tapers slightly, outer tube does not. Sclerotized piece can be seen extended from distal end of outer tube (Fig. 2A). Accessory piece absent. Vitellarium extensive throughout body, extended from level of pharynx to posterior level of intestinal caeca (Figs. 1 and 3A). Most reproductive and other internal structures throughout middle body not defined through vitellarium.

#### 3.1.2 Remarks

Based on body shape and the comparative morphology of the haptoral structures, squamodisc and male copulatory organ, *Diplectanum timorcanthus* n. sp*.* most closely resembles *D. oliveri* and *D. gladulosum* , both parasitising the mulloway *Argyrosomus hololepidotus* (Lacepède, 1802) (Sciaenidae) from the Swan River Estuary and Cockburn Sound, Perth, Western Australia (Williams 1989) (Table 2). *Diplectanum timorcanthus* n. sp. can be differentiated from *D. oliveri* by possessing a much shorter MCO, and with the central tube often erect from the curved distal margins of the outer tube. The distal end of the MCO from *D. timorcanthus* n. sp. also tapers slightly, while the MCO of *D. oliveri* does not and is rather spatulate at the distal end. Despite similarities in the MCO structure between *D. timorcanthus* n. sp. and *D. glandulosum,* the body length of *D. timorcanthus* n. sp. is much greater and the ventral bar groove less conspicuous, when compared with *D. glandulosum.*

**Fig. 4:**
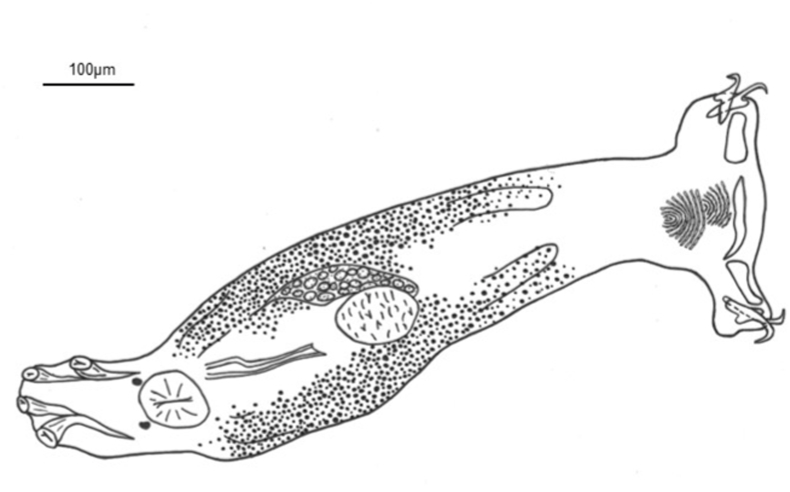
Composite drawing of *Diplectanum diacanthi* n. sp.

**Fig. 5:**
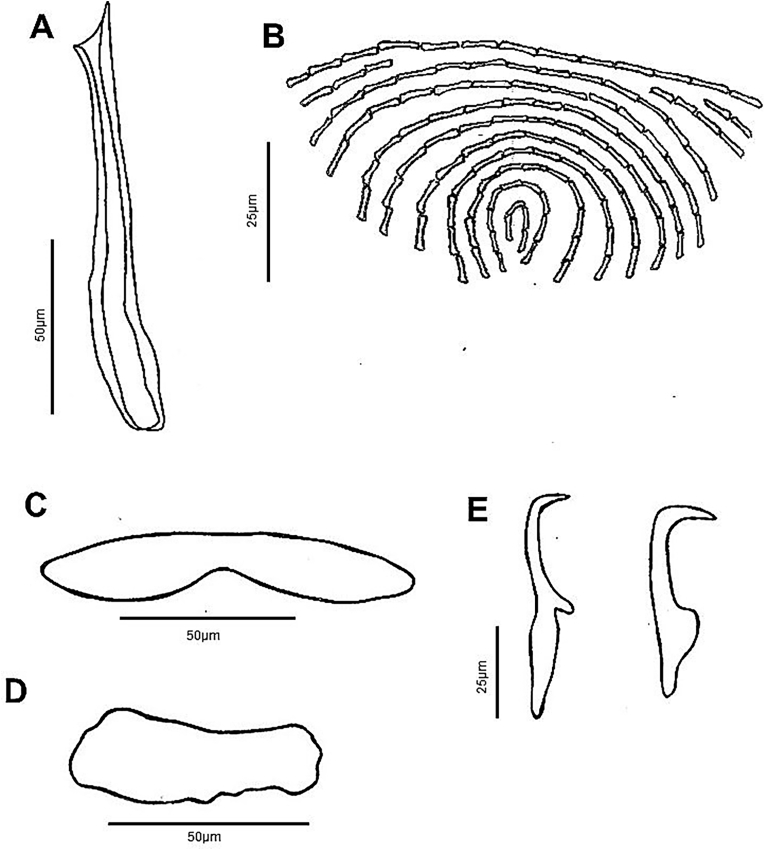
*Diplectanum diacanthi* n. sp. characters of morphological significance. A, MCO; B, Squamodisc; C, Ventral Bar; D, Dorsal Bar; E, Ventral and Dorsal Anchors.

***Diplectanum diacanthi* n. sp.** (Fig. 3B, D, F, Fig. 4, Fig. 5)

*Type host: Protonibea diacanthus* (Lacepède, 1802) (Sciaenidae) ‘Black Jewfish’ or ‘Black-spotted croaker’.

*Type locality:* Caution Point, Beagle Gulf, Northern Territory, Australia. Other localities include Van Diemen Gulf, Timor Sea, Northern Territory, Australia.

*Site in host:* Gill filaments.

*Type-material:* Holotype: MAGNT D001922; Paratypes: MAGNT D001923, QM G240633, WAM V11010

*GenBank accession:* 28S (OQ846932-OQ846934), ITS1 (OQ846938-OQ846939)

*Etymology:* The specific name is derived from the host species from which the type specimen of *Diplectanum diacanthi* n. sp. was collected.

#### 3.1.3 Description

Based on 22 specimens. Body medially rounded, fusiform, with smooth tegument; total length 1,087 (645–1,870); greatest width 253 (127-460), near level of testis. Four cephalic lobes moderately defined. Two pairs of head organs, 15 (10-24) long, 13 (9-21) wide, moderately developed as cephalic lobes, adjacent cephalic areas. Cephalic glands poorly defined, extending towards pharynx. Eye spots 2 when visible, poorly developed or represented by scattered granules, dorsal, anterior to pharynx. Mouth subterminal; pharynx bulbous, length 64 (38-101), width 62 (27-95). Oesophagus short to absent. Intestinal caeca bifurcates short distance posterior to pharynx, crura blind posteriorly. Peduncle slightly tapered posteriorly, 168 (89-366) long, 96 (54-163) wide, peduncular spines absent. Haptor laterally expanded (Figs. 3B), 67 (51-101) long, 235 (151-301) wide. Squamodiscs subtriangular in shape, ventral and dorsal (Fig. 3D); each formed with approximately 12 rows of sclerotized dumbbell-shaped rods, anterior rows concentric, progressively tending to form straight line posteriorly (Fig. 5B); squamodisc 48 (23-74) long, 65 (35-101) wide. Anchors 2 pairs, ventral and dorsal (Fig. 5E). Ventral anchor length 60 (46-75), anchor base width 10 (4-20); anchor with elongate, slightly tapered deep root, longer than superficial root, knob-like superficial root with rounded point, curved shaft, short point (Fig. 5E). Dorsal anchor length 66 (46-78), anchor base width 10 (4-14); anchor with wide tapered deep root, incipient superficial root, straight shaft, short point (Fig. 5E). Hooks not observed. Ventral bar with tapering, rounded ends, constricted midregion, ventral longitudinal groove (Figs. 5C), 109 (93-124) long, greatest width 16 (8-19). Paired dorsal bar, 61 (48-66) long, greatest width 20 (12-40); not overlapping each other, medial end expanding spatulate wider than lateral end, medial end often just overlapping lateral points of ventral bar, some cases overlapping with ventral bar absent (Figs. 3F, 4 and 5D). Male copulatory organ (MCO) sclerotized, elongate (Figs. 5A), 96 (81-110) long, greatest width 10 (9-12); composed of two nested tubes which appear to be joined at the distal end. MCO relatively straight, inner tube tapers slightly. Distal end bifurcates into two-pointed fork like expansion. Distal end of sclerotized piece curves concave between the two points (Fig. 5A). Accessory piece absent. Vitellarium well developed, most dense anteriorly from level of pharynx and extending posteriorly to anterior limit of peduncle (Figs. 3B and 4). Most reproductive and other internal structures throughout middle body not defined through vitellarium.

#### 3.1.4 Remarks

The body shape of *Diplectanum diacanthi* n. sp. slightly resembles those described from *D. timorcanthus* n. sp. and *D. oliveri*, however the morphological resemblance ceases from this point. Of the haptoral structures, the ventral bar of *D. diacanthi* n. sp. is much narrower than others described. The morphology of the dorsal bars and the squamodisc are in fact more similar to those from the type species *Diplectanum aequans* (Wagener, 1857) Diesing, 1858*.* The dorsal bars of these species are much shorter and wider and possess subtriangular ventral and dorsal squamodiscs with progressively straightening rows of rodlets at the posterior end. *D. aequans* does however exhibit distal tapering of the dorsal bars and a larger number of rows of the squamodisc, differentiating the type species to *D. diacanthi* n. sp.

### 3.2 Molecular analyses

**Fig. 6:**
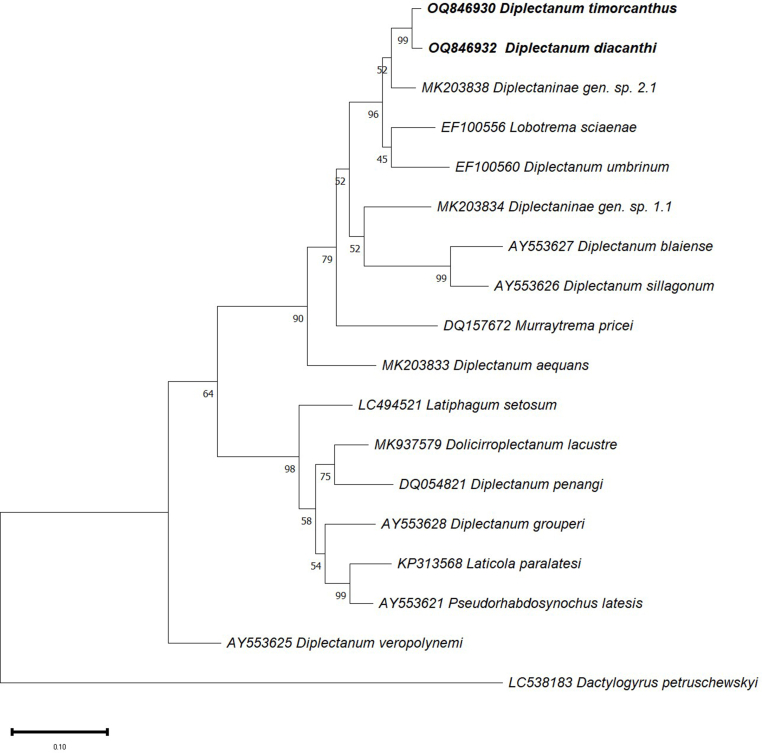
Phylogenetic tree based on the Tamura-Nei model for sequences of the 28S rDNA (partial) gene region of *Diplectanum timorcanthus* and *Diplectanum diacanthi* (bold) in this study, with closely related sequences available from the GenBank database (Table 1). Bootstrap values are labelled alongside each node.

**Fig. 7:**
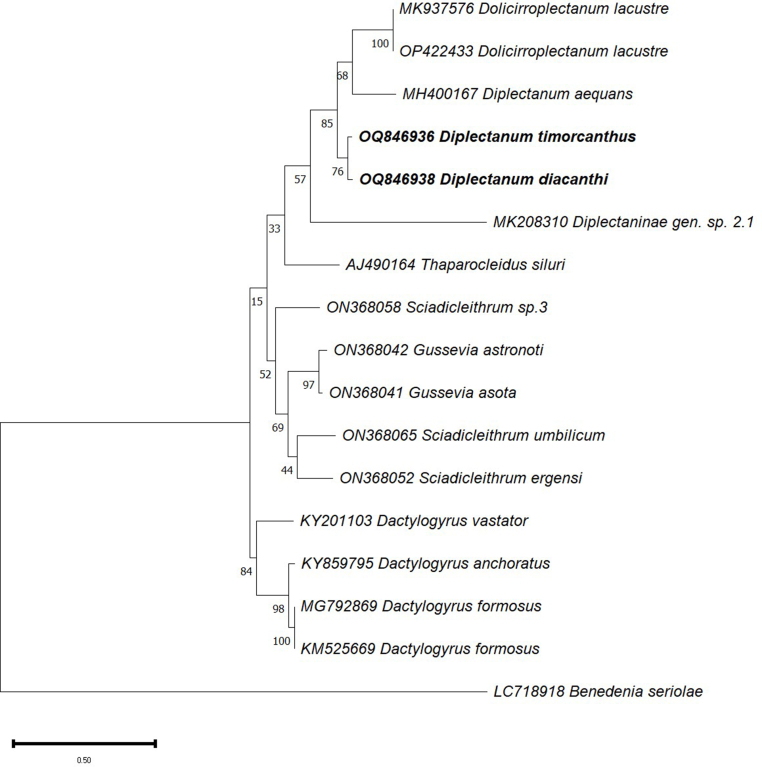
Phylogenetic tree based on the Tamura-Nei model for sequences of the ITS1 region of *Diplectanum timorcanthus* and *Diplectanum diacanthi* (bold) in this study, with closely related sequences available from the GenBank database (Table 1). Bootstrap values are labelled alongside each node.

Phylogenetic analysis of the nuclear 28S rDNA and ITS1 sequences was performed using the Maximum Likelihood analysis in MEGA version 11 (Fig. 6, Fig. 7). Sequences of the 28S region (Fig. 6) separated into two distinct clades, with the upper region of the tree rooted by the type species *D. aequans*. All specimens rooted by *D. aequans* represent diplectanids from sciaenid hosts and based on the most up to date morphological key (Domingues and Boeger 2008), *D. timorcanthus* n. sp. and *D. diacanthi* n. sp. belong to the *Diplectanum* genus. The two species from this study, *D. timorcanthus* n. sp. and *D. diacanthi* n. sp. grouped within a single clade, with a bootstrap confidence value of 99% (Fig. 6). Although not supported by a very strong posterior probability, both *D. timorcanthus* n. sp. and *D. diacanthi* n. sp. clustered with Diplectaninae gen. sp. 2.1. Branched from the same node with a strong bootstrap value are the two specimens *Lobotrema sciaenae* (Bychowsky and Nagibina, 1977) Oliver, 1987 and *Diplectanum [sensu lato] umbrinum* Tripathi, 1959, both described as coming from a sciaenid host. The remaining 28S sequences of species of *Diplectanum* in Fig. 6 do not group with species from this study, however since their deposition in GenBank, these species have been redescribed and are no longer classified as *Diplectanum*.

The same specimens were sequenced at the ITS1 region with *D. timorcanthus* n. sp. and *D. diacanthi* n. sp. falling within a single clade (Fig. 7). Apart from the two new species described in this study, Diplectaninae gen. sp. 2.1 represents the only other published sequence of a diplectanid monogenean from a sciaenid host. Branching from the same node and forming a separate clade from *D. timorcanthus* n. sp. and *D. diacanthi* n. sp., are the type species *D. aequans* and two identical sequences of *Dolicirroplectanum lacustre* (Thurston & Paperna, 1969) Kmentová et al., 2020, collected from Latidae hosts. The posterior probabilities across the nodes in Fig. 7 consistently provide low support for the phylogenetic relationships shown.

## 4 Discussion

This study describes two new species, *Diplectanum timorcanthus* n. sp. and *Diplectanum diacanthi* n. sp. parasitising *P. diacanthus* in Australian waters, using a combined morphological and molecular approach. These are new host and geographical records for genus *Diplectanum.* Taxonomy for members of the Diplectanidae is generally based on morphology alone, with very few species descriptions utilising a combination of morphological and molecular data. Consequently, the phylogenetic relationships within the *Diplectanum* genus remain largely unexplored (Villar-Torres et al., 2019). The existing molecular phylogeny of *Diplectanum* includes only sequences for the type species *D. aequans,* and three species of *Diplectanum* considered *incertae sedis* by Domingues and Boeger (2008). Phylogenetic analysis of the Diplectanidae by Villar-Torres et al. (2019) revealed an association between diplectanid clades and specificity to a host at the family level, with taxa parasitising the same fish family often clustering together (Villar-Torres et al., 2019). This is reflected in the results from the present study with specimens in the 28S tree (Fig. 6) separating into two distinct clades, with diplectanids from sciaenid hosts explicitly making up the upper clade. The new species *D. timorcanthus* n. sp. and *D. diacanthi* n. sp. group within a single clade, and along with the remaining diplectanids from sciaenid hosts, branch with high support from the type species *D. aequans.* Based on the phylogenetic position and the most up to date morphological key, both new species described here are described as species of *Diplectanum*. Within the upper clade of the 28S tree, Diplectaninae sp. 2.1, which was collected from a sciaenid species in the western Mediterranean Sea, branches from the same node as the new described species, along with both *L. sciaenae* and *D. umbrinum* which also form part of this upper cluster (Villar-Torres et al., 2019). The descriptions of the *L. sciaenae* and *D. umbrinum* specimens from which molecular sequences were obtained were not published, and without any corresponding morphological descriptions (Table 1) or designated collection location and host species, are both potentially misidentified. Therefore, for the purpose of this study it is assumed that the “*L. sciaenae*” and “*D. umbrinum*” specimens were collected from a sciaenid host and as a result, have fallen within this upper clade. The clustering pattern was also relevant to the monogeneans in Villar-Torres et al. (2019), with one genetically distinct species of Diplectanidae (Diplectaninae sp. 2.1) from *Umbrina* sp., recovered as a sister taxa to *Lobotrema sciaenae* and *Diplectanum [sensu lato] umbrinum,* which were both Indo-Pacific parasites of sciaenid hosts (Villar-Torres et al., 2019). The remaining specimens forming the upper clade in Fig. 6 should also be treated with caution given both *Paradiplectanum blairense* (Gupta and Khanna, 1974) Domingues and Boeger (2008) and *Paradiplectanum sillagonum* (Tripathi, 1959) Domingues and Boeger (2008) have been redesignated to a different genus (Table 1), and *Murraytrema pricei* Bychowsky and Nagibina (1977) has been described solely from phylogeny and not morphology. Further highlighting the fluidity of the Diplectanidae taxonomy, specimens grouped in the bottom clade, although originally described as *Diplectanum* (Table 1), have since been redescribed and can therefore be classified as being distantly related to *D. timorcanthus* n. sp. and *D. diacanthi* n. sp.

The phylogenetic analysis of the internal transcribed spacer (ITS1) region did not contribute significantly to the taxonomic framework of the Diplectanidae in this study given the lack of sequences in GenBank (Table 1, Fig. 7). The new species *D. timorcanthus* n. sp. and *D. diacanthi* n. sp. formed a clade with relatively high support with the type species *D. aequans* and identical specimens of *D. lacustre,* collected from Moronidae fishes and Latidae fishes respectively. The only other specimen from a sciaenid host was Diplectanidae sp. 2.1 and this species formed the root of the clade of specimens in which *D. timorcanthus* n. sp. and *D. diacanthi* n. sp. clustered. Better taxonomic relationships within the Diplectanidae will be found when more sequences are published, and perhaps when many described species are re-examined in light of new molecular data.

The morphological boundaries within *Diplectanum* are not well established and species within this genus exhibit a high degree of variability, including the presence or absence of an accessory piece, position of the vaginal aperture and morphology of the copulatory complex (among others) (Mendoza Franco et al., 2008). For this reason, Kritsky et al. (2000), and recently Domingues and Boeger (2008), rejected the monophyly of species of *Diplectanum* which was attributed to the absence of morphological boundaries to delimit the species included in the genus (Villar-Torres et al., 2019). After morphological and cladistic analysis the *Diplectanum* genus was restricted to species possessing the combination of the following characteristics: (1) male copulatory organ formed by two nested tubes; (2) accessory copulatory organ; (3) prostatic reservoir separated into three zones; (4) two squamodiscs (of which are considered accessory adhesive organs) (Domingues and Boeger 2008). Species differentiation within the Diplectanidae has often relied on the interspecific variation of MCO morphology, however in some instances it is the combination of this differentiation with a number of other morphological characters that confirms the rise of new species, as is the case for the descriptions of *D. timorcanthus* n. sp. and *D. diacanthi* n. sp*.*

In the cladistic analysis by Domingues and Boeger (2008), *Diplectanum* appears a sister taxa of *Lobotrema*, within Diplectaninae as indicated by the sharing of accessory copulatory organ, and prostatic reservoir separated into three zones. Bychowsky and Nagibina (1977) suggested that these two taxa, along with *Murraytrema* are closely related. The two new species described in this study possess only the combination of characteristics that align with those features characteristic of the *Diplectanum,* as both species belonging to *Lobotrema* and *Murraytrema* do not possess an accessory adhesive organ (Domingues and Boeger 2008). Within the genus, *Diplectanum oliveri* and *Diplectanum glandulosum* represent those species most morphologically similar to *Diplectanum timorcanthus* n. sp. (Williams 1989), with the newly described *Diplectanum diacanthi* n. sp. seemingly most dissimilar to existing species of *Diplectanum* from Australia. The only other report of species of *Diplectanum* from northern Australia (Young 1969), with the description of several new species from marine and freshwater locations at Heron Island, Green Island and Moreton Bay of Queensland, were found to be morphologically dissimilar to *Diplectanum* spp. and have since been reclassified as species of the *Pseudorhabdosynochus* Yamaguti, 1958, and *Echinoplectanum* Justine and Euzet, 2006. Additionally, the original reports from Young (1969) also described monogeneans from Serranidae hosts and not sciaenids. Therefore, Australian reports of *Diplectanum* still remain scarce.

## Funding

This project was supported by the 10.13039/501100000979Fisheries Research and Development Corporation (#2018–027) in collaboration with 10.13039/501100001803Charles Darwin University and 10.13039/100008344Australian Institute of Marine Science. Megan Porter was supported by a 10.13039/501100001769Charles Sturt University AGRTP Scholarship.

## Availability of data and material

All data produced for this study are provided in the manuscript.

## Authors contribution

All authors contributed to the study conception and design. Funding acquisition, Investigation, Methodology, Data curation, and Writing: Megan Porter; Writing review and editing, Investigation, and Supervision: Dr Diane P. Barton; Methodology, Data curation, Writing review and editing: Dr Nidhish Francis; Writing review and editing, and Supervision: Professor Shokoofeh Shamsi.

## Ethics approval

Ethics approval for this study was provided by the Charles Darwin University (CDU) Animal Ethics Committee (AEC), approval number #A19009.

## Consent to participate

Not applicable.

## Consent for publication

Not applicable.

## Declaration of competing interest

The authors declare that they have no known competing financial interests or personal relationships that could have appeared to influence the work reported in this paper.
